# Xiburema Virus, a Hitherto Undescribed Virus within the Family *Rhabdoviridae* Isolated in the Brazilian Amazon Region

**DOI:** 10.1128/genomeA.00454-14

**Published:** 2014-06-19

**Authors:** Ana Lucia M. Wanzeller, Lívia C. Martins, José Antonio P. Diniz Júnior, Daniele Barbosa de Almeida Medeiros, Jedson F. Cardoso, Daisy E. A. da Silva, Layanna F. de Oliveira, Janaina M. de Vasconcelos, Márcio R. T. Nunes, João Lídio da S. G. Vianez Júnior, Pedro F. C. Vasconcelos

**Affiliations:** a Department of Virology, Evandro Chagas Institute, Ministry of Health, Ananindeua, Brazil; b Department of Arbovirology and Hemorrhagic Fevers, Evandro Chagas Institute, Ministry of Health, Ananindeua, Brazil; c Department of Electron Microscopy, Evandro Chagas Institute, Ministry of Health, Belém, Brazil; d Center for Technological Innovation, Evandro Chagas Institute, Ministry of Health, Ananindeua, Brazil

## Abstract

We report here the first complete open reading frame (ORF) genome sequence of Xiburema virus (XIBV), that of strain BE AR362159, isolated from mosquitoes (*Sabethes intermedius*) in Sena Madureira, Acre state, northern Brazil. All genes showed similarities with those belonging to members of the family *Rhabdoviridae*.

## GENOME ANNOUNCEMENT

The family *Rhabdoviridae* is a large family of viruses comprising >46 viruses distributed in six recognized genera (*Vesiculovirus, Lyssavirus, Ephemerovirus, Novirhabdovirus, Cytorhabdovirus*, and *Nucleorhabdovirus*) (1), five species unclassified in a genus, and 150 ungrouped viruses (1). They are bullet-shaped enveloped viruses with a mean diameter of 45 to 100 nm and lengths of 100 to 430 nm. The genome is a unique single-stranded negative-sense RNA (ssRNA^−^) possessing five main polycistronic genes: those for nucleocapsid (N), phosphoprotein (P), matrix (M), glycoprotein (G), and polymerase (L) (2). Some rhabdoviruses present accessory genes between the main genes, but their functions remain to be determined (3). Intergenic regions, as well as terminal noncoding regions (3′ and 5′ noncoding regions [NCRs]), are also present (2, 4).

Viruses in this family infect plants and animals, but only species from the genera *Vesiculovirus*, *Lyssavirus*, and *Ephemerovirus* cause diseases in mammals. An exception is the sigma virus (type species *Drosophila melanogaster* sigma virus [DmelSV]), which is transmitted vertically among parent flies (i.e., through both eggs and sperm) (5). The vectors for vesiculoviruses and ephemeroviruses are known arthropods and include mosquitoes, sandflies, ticks, and midges (6), while for lyssaviruses, they are wild mammals. Rabies virus (RABV), vesicular stomatitis virus (VSV), and bovine ephemeral fever virus (BEFV) are the type species for the *Lyssavirus*, *Vesiculovirus*, and *Ephemerovirus* genera, respectively, and also the most important rhabdoviruses in terms of public health (7–9).

Xiburema virus (XIBV) (strain BE AR362159) was isolated from mosquitoes (*Sabethes intermedius*) in the municipality of Sena Madureira, Acre state, northern Brazil (09°3′S, 68°39′W) in 1974. Complement fixation and neutralization tests were used to antigenically characterize and define Xiburema virus as an ungrouped virus. Transmission electronic microscopy (TEM) was also used to confirm the bullet-shaped nature of the virions, as previously described, placing it as a member of the *Rhabdoviridae* (10).

The XIBV particles were precipitated using polyethylene glycol (PEG) centrifugation, as previously described (8), and the supernatant was treated with DNase and RNase (Ambion) for host contaminant removal. The treated samples were then used for RNA extraction and full-length genome sequencing using a combination of sequencing platforms, the GS FLX 454 (Roche, Life Sciences) and Ion Torrent PGM (Life Technologies). Regardless of the sequencing platform used, the method used to obtain the genome basically corresponded to the following steps: RNA fragmentation, library preparation (cDNA), emulsion PCR, and sequencing, as previously described (11, 12). The sequencing steps were carried out at the Genomic Core of the Center for Technological Innovation, Evandro Chagas Institute, Brazilian Ministry of Health, Ananindeua, Brazil.

The genome was obtained by employing a *de novo* hybrid assembly strategy using both Ion Torrent and GS FLX 454 reads simultaneously with the software Mira 4.0. Visual inspection was performed with the software Geneious version 6.1.4. The total genome recovered was 12,240 nucleotides (nt) in length, with a mean coverage of 383-fold. The five main genes 3′-N-P-M-G-L-5' were recognized, as well as two putative accessory genes (pAG1 and pAG2) between the G and L genes.

This is the first report of the complete genome sequence for Xiburema virus, an ungrouped Brazilian rhabdovirus.

### Nucleotide sequence accession number.

The complete genome sequence has been deposited in GenBank under the accession no. KJ636781.
